# Enhancing Wheat Spike Counting and Disease Detection Using a Probability Density Attention Mechanism in Deep Learning Models for Precision Agriculture

**DOI:** 10.3390/plants13243462

**Published:** 2024-12-11

**Authors:** Ruiheng Li, Wenjie Hong, Ruiming Wu, Yan Wang, Xiaohan Wu, Zhongtian Shi, Yifei Xu, Zixu Han, Chunli Lv

**Affiliations:** China Agricultural University, Beijing 100083, China

**Keywords:** wheat, spike, disease detection, smart agriculture, deep learning

## Abstract

This study aims to improve the precision of wheat spike counting and disease detection, exploring the application of deep learning in the agricultural sector. Addressing the shortcomings of traditional detection methods, we propose an advanced feature extraction strategy and a model based on the probability density attention mechanism, designed to more effectively handle feature extraction in complex backgrounds and dense areas. Through comparative experiments with various advanced models, we comprehensively evaluate the performance of our model. In the disease detection task, our model performs excellently, achieving a precision of 0.93, a recall of 0.89, an accuracy of 0.91, and an mAP of 0.90. By introducing the density loss function, we are able to effectively improve the detection accuracy when dealing with high-density regions. In the wheat spike counting task, the model similarly demonstrates a strong performance, with a precision of 0.91, a recall of 0.88, an accuracy of 0.90, and an mAP of 0.90, further validating its effectiveness. Furthermore, this paper also conducts ablation experiments on different loss functions. The results of this research provide a new method for wheat spike counting and disease detection, fully reflecting the application value of deep learning in precision agriculture. By combining the probability density attention mechanism and the density loss function, the proposed model significantly improves the detection accuracy and efficiency, offering important references for future related research.

## 1. Introduction

With the continuous growth in the global population, the issue of food security has become increasingly prominent, making efficient and precise agricultural technologies key factors in driving the development of modern agriculture [1,2,3]. Among these, wheat spike counting and disease detection are important agricultural monitoring tasks that hold significant importance for improving crop yields, optimizing agricultural resource allocation, and the early diagnosis and treatment of diseases [4,5,6]. Traditionally, these tasks have relied on manual observation and statistics, which are not only time-consuming and labor-intensive but also subject to subjective judgment, resulting in lower accuracy and efficiency [7,8].

Wheat spike counting serves as a critical indicator for yield prediction and provides essential data for the rational allocation of agricultural resources, whereas disease detection is crucial for ensuring crop health and stabilizing yields. By simultaneously monitoring these aspects, more comprehensive information can be obtained to support agricultural decision making. For instance, understanding wheat spike distributions in regions affected by concentrated disease outbreaks can aid in pinpointing hotspots and implementing precise interventions. Additionally, combining disease detection data with spike density analysis can offer insights into the health status of wheat spikes in different areas, optimizing crop management strategies. Therefore, integrating these two tasks enhances the overall efficiency of agricultural monitoring and fosters multidimensional data analysis.

Wheat spike counting is a crucial indicator for assessing wheat yield. By accurately counting, it can effectively predict crop yields, significantly impacting agricultural production management and harvest forecasting [9,10]. However, due to variations in the size, shape, and maturity of wheat spikes, along with the complexity of field environments—such as lighting conditions and occlusions between spikes—accurate wheat spike counting becomes a challenge. With the rapid development of computer vision technology, especially the application of deep learning, new technical means have emerged for precise wheat spike counting and disease detection [11,12]. Deep learning models, particularly convolutional neural networks (CNNs), have shown exceptional performance in tasks like image classification, object detection, and semantic segmentation due to their powerful feature extraction capabilities [13,14]. For example, Shen et al. [15] proposed a lightweight wheat spike detection and counting method based on YOLOv5s. This method utilizes the ShuffleNetV2 lightweight convolutional neural network, optimizing the YOLOv5s model by reducing the number of parameters and simplifying the complexity of the computation process. Additionally, a lightweight upsampling operator was introduced in the feature pyramid structure to perceptually reorganize features, mitigating the impact of the lightweight process on the model detection performance. The experimental results show that the model’s mean average precision (mAP) can reach 94.8%, but there remains room for optimization and the potential application of other acceleration technologies to improve the inference and detection speed. Yang et al. [16] proposed an automatic wheat spike localization and counting method based on FIDMT-GhostNet to address challenges like complex backgrounds, dense spikes, small target sizes, and varying spike sizes. To tackle the issue of less feature information caused by small wheat spikes, they introduced a dense upsampling convolution module to enhance the image resolution and extract more detailed information. Finally, to overcome background noise or wheat spike interference, they designed a local maximum detection strategy for automated spike counting. Experimental results indicated an accuracy of 0.9145 for the wheat spike counting model, although storage usage and computational demands remain concerns.

The early detection of wheat diseases is particularly critical for the formulation of prevention and control strategies, and for reducing crop losses. Traditional disease detection methods often rely on the experience of agricultural experts and periodic field inspections, which are not only inefficient but also difficult to implement on a large scale for disease monitoring [17,18]. In recent years, deep learning technologies have demonstrated outstanding performance in the field of image recognition [19]. These models can automatically extract and process image features, greatly enhancing the accuracy and efficiency of crop recognition. Biswas Srabani et al. [20] proposed a new energy-efficient convolutional neural network architecture and compared it with two existing models, VGG19 and Inception V3. The results indicated that their proposed model achieved an accuracy of 95.17%. To address the challenge of detecting wheat diseases in complex fields, Fang et al. [21] constructed a lightweight multi-scale CNN model that integrates CBAM and ECA modules into residual blocks, enhancing the model’s focus on diseases and reducing the influence of complex backgrounds on disease identification. Their proposed method achieved an accuracy of 98.7% on the test dataset, outperforming classical convolutional neural networks such as AlexNet, VGG16, and Inception ResNet V2; however, the current crop disease image database is not comprehensive, and the image quality is often poor. Kumar Varun et al. [22] combined random forests and convolutional neural networks to classify eight common wheat diseases, and experiments showed that this hybrid model achieved an accuracy of 95%, indicating its effectiveness in enhancing agricultural yield and ensuring the global food supply.

This study proposes a probability density-based attention mechanism model for precise wheat spike counting and disease detection. Unlike traditional deep learning methods, our model introduces a probability density model to better handle the occlusion issues between wheat spikes and utilizes an attention mechanism to optimize the areas of focus in the model, thereby improving the detection accuracy and robustness. The innovations of this study are as follows:

- Probability Density-Based Attention Mechanism: To address the challenges of dense overlap and complex backgrounds in wheat spikes, a probability density-based attention mechanism is proposed. This mechanism highlights key feature regions in images and reduces the background noise interference, thereby improving the counting accuracy and robustness.
- Transformer-Based Object Detection Architecture: A Transformer architecture tailored for object detection tasks is introduced, suitable for processing large-scale, high-resolution image data. Compared to traditional CNNs, the Transformer excels at capturing long-range dependencies in images, showing significant advantages in handling complex agricultural scenarios such as varying lighting conditions and occlusions.
- Customized Density Loss Function: A density loss function specifically designed for agricultural image processing is developed to optimize the model’s performance in wheat spike counting and disease detection tasks. This function effectively handles both dense and sparse regions while enhancing the model’s sensitivity to disease features, enabling more accurate assessments of wheat health and yield.

This study aims to improve the accuracy of wheat spike counting and disease detection, exploring the potential of deep learning technology in the agricultural field. By proposing a model that combines a probability density-based attention mechanism and a density loss function, we address the challenges of feature extraction in complex backgrounds and densely populated areas, providing a novel approach to image processing technology in precision agriculture.

## 2. Related Work

### 2.1. Probability Density Model

In the field of computer vision, particularly in crowd counting and agricultural image analysis such as wheat spike counting, probability density models provide an effective solution for accurately predicting and analyzing the number of targets in complex scenes [23,24,25]. In computer vision, probability density models predict the total number of targets in an image by estimating the density of targets at each pixel [26,27]. This method is especially suited for applications like crowd counting, where the number of targets (such as heads or wheat spikes) is large and may be partially obscured. The fundamental assumption of the model is that each target in the image can generate a density peak at its corresponding location, and this effect is simulated by placing a Gaussian kernel at the target locations, thus generating a continuous density map across the entire image [28,29]. Specifically, for an image containing *N* targets, each target location is marked as $\left(x_{i},y_{i}\right)$, and Gaussian kernels are placed at these locations; the density map D(x,y) of the entire image can be calculated using the following formula:

(1) $$D\left(x,y\right)=\sum_{i=1}^{N}\delta \left(x-x_{i},y-y_{i}\right)*G_{\sigma }\left(x,y\right)$$

Here, $\delta (x-x_{i},y-y_{i})$ is the Dirac function, indicating a target at the position $\left(x_{i},y_{i}\right)$, and $G_{\sigma }\left(x,y\right)$ is a two-dimensional Gaussian function with standard deviation σ, used to simulate the impact of each target on the surrounding pixels. By adjusting the size of σ, the smoothing level of the Gaussian kernel can be controlled, thereby adapting to the actual distribution of targets in the image. Applying the probability density model to wheat spike counting effectively addresses common issues in wheat field image analysis, such as partial occlusion and the dense distribution of wheat spikes [30,31]. Initially, wheat field images are preprocessed to identify and mark the position of each wheat spike. Then, using Gaussian kernels, density peaks are generated at each marked position, and the resulting density map not only reflects the distribution of wheat spikes but can also be used to estimate the total number of wheat spikes in the field.

Moreover, occlusion is a particular challenge in wheat spike counting. Wheat spikes often overlap each other due to wind or growth patterns, and traditional detection methods may not accurately distinguish touching or partially occluded wheat spikes [32,33]. The probability density model effectively handles this situation because it does not simply count but estimates the number of wheat spikes covering each pixel to infer the total count, significantly enhancing the model’s adaptability to complex scenes [34]. Further integrating a probability density-based attention mechanism into the wheat spike counting model allows the model to focus more on areas densely packed with wheat spikes. This attention mechanism dynamically adjusts the model’s focus by analyzing the density map, prioritizing areas likely to contain more wheat spikes [35]. For example, the probability weight of each pixel in the density map can be calculated to guide the model in focusing more on these areas during feature extraction and subsequent processing.

(2) $$\alpha \left(x,y\right)=\frac{\exp(D(x,y))}{\sum_{x^{\prime },y^{\prime }}\exp\left(D\left(x^{\prime },y^{\prime }\right)\right)}$$

Here, α(x,y) is the attention weight calculated from the density map D(x,y), ensuring that the model can adaptively adjust its focus on different areas, especially in situations where wheat spike density varies significantly [36]. By combining the probability density model with a probability density-based attention mechanism, this study not only enhances the accuracy and efficiency of wheat spike counting but also provides new approaches and tools for handling other similar agricultural image analysis tasks.

### 2.2. Attention Mechanism

The attention mechanism was first widely applied in the field of natural language processing (NLP), particularly in tasks such as machine translation and text understanding [37,38,39]. In image recognition tasks, the attention mechanism effectively helps models focus on important areas within an image, thereby significantly enhancing performance in classification and detection [40,41]. Specifically, the computation of the attention mechanism can be expressed by the following formula:

(3) $$\mathrm{Attention}\left(Q,K,V\right)=\mathrm{softmax}\left(\frac{QK^{T}}{\sqrt{d_{k}}}\right)V$$

Here, *Q*, *K*, and *V* represent the query, key, and value, respectively, and $d_{k}$ is the dimension of the key. This formula calculates the similarity scores between the query and all keys, then normalizes these scores to produce a distribution of weights. These weights are subsequently used to weight the corresponding values, thus focusing the model’s attention. In the field of image processing, particularly in tasks such as object detection and image classification, the attention mechanism has also shown great potential [42,43]. This mechanism, by recalibrating the response of the channels, enhances the model’s perception of useful features while suppressing unimportant ones. In this study, we apply the attention mechanism to a probability density-based model for wheat spike counting and disease detection, especially for dealing with dense and partially obscured wheat spikes in agricultural images [44,45]. For this, we have designed a probability density-based attention layer aimed at enhancing the model’s perception and processing capabilities for densely packed wheat spike areas. The computation of this attention layer can be expressed as follows:

(4) $$A\left(x,y\right)=\mathrm{softmax}\left(\beta \cdot \log(D(x,y)+\epsilon )\right)$$

Here, D(x,y) represents the density estimation map obtained from the probability density model, β is a trainable scaling parameter, and ϵ is a small constant used to ensure numerical stability. Through this approach, the model not only focuses more on areas with a high density of wheat spikes but can also more effectively distinguish wheat spikes affected by diseases, as diseases often alter the appearance and distribution of the spikes [46].

## 3. Materials and Method

### 3.1. Dataset Collection

**Figure 1:**
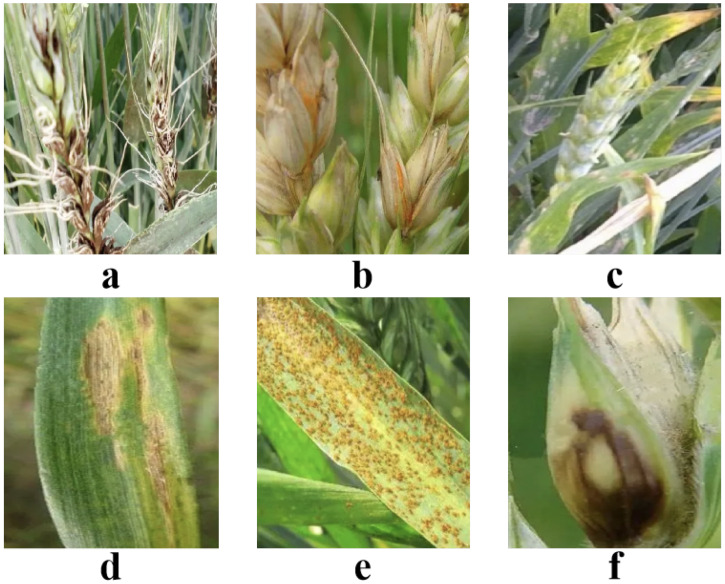
Dataset samples. (**a**) is loose smut, (**b**) is fusarium head blight, (**c**) is powdery mildew, (**d**) is leaf spot, (**e**) is glume blotch, (**f**) is rust.

In this study, image collection is a crucial step, and our images are primarily sourced from the Science Park of the West Campus of China Agricultural University in Haidian District, Beijing, along with some legally authorized online images. These online images are obtained from the Kaggle platform, specifically the wheat spike dataset [47], which is widely used in deep learning research related to wheat spike counting and disease detection. This dataset provides diverse and high-quality images with precise annotations, including various field conditions, lighting environments, and complex backgrounds, making it highly suitable for our study’s objectives. This selection of the collection area is based on its geographical advantage as a hub for agricultural research, providing abundant crop samples and disease manifestations. Additionally, the introduction of online images aims to expand the sample size, ensuring stronger generalization capability during model training, as shown in Figure 1.

#### 3.1.1. Disease Image Collection

During our field collection in the Science Park, we selected well-growing wheat plants to better observe and document their disease characteristics. Our collection equipment included a high-resolution digital SLR camera, specifically the Canon EOS 90D, equipped with a 75–300 mm zoom lens suitable for agricultural scene photography, which maintains high image clarity at different distances. With a resolution of 32 million pixels, the camera can capture subtle disease features, providing a solid foundation for subsequent image processing and analysis. During the collection process, we ensured that there were enough image samples for each type of disease, particularly focusing on common diseases such as loose smut, fusarium head blight, powdery mildew, leaf spot, glume blotch, and rust. The number of images for each disease ranged between 1000 and 2000, as detailed in Table 1.

When collecting disease images, we paid special attention to the appearance characteristics of various diseases to provide accurate samples for subsequent model training. We ensured that samples covered multiple wheat growth stages, including the seedling, jointing, heading, and grain filling periods, to comprehensively capture disease characteristics and progression. First, loose smut is a common disease in wheat, characterized by small black dots on the surface of the spikes, which are actually spore sacs of the pathogen. These spore sacs are typically deep black, measuring about 1 to 2 mm in diameter, and they can reflect light in sunlight, making them particularly noticeable against the green spikes. As the disease progresses, the spikes may become weaker and may even dry out before maturity, leading to significant declines in both yield and quality. Loose smut was mainly observed and documented during the heading and early grain filling stages when symptoms were most apparent.

Fusarium head blight presents more complex symptoms. Affected spikes often exhibit softening, becoming loose and lacking elasticity, and, upon touch, one can feel the surface becoming increasingly sticky, forming a reddish or pinkish mold layer. This mold layer not only makes the wheat grains difficult to shed but also emits a characteristic moldy smell that affects their edibility. Fusarium head blight is particularly severe in humid weather, typically occurring during the wheat grain filling period, so we specifically collected samples during this critical stage, ensuring that conditions such as humidity and temperature were accurately recorded to represent its peak severity. Powdery mildew is characterized by a white powdery substance on the surface of wheat leaves, which consists of the pathogen’s spores. In the early stages of the disease, small white spots appear on the leaf surfaces, which gradually enlarge as the condition worsens, eventually forming a thick white mold layer that can lead to yellowing and even drying of the leaves in severe cases. For powdery mildew, we documented its development across the seedling, jointing, and heading stages, carefully observing and photographing the distribution and density of the mold layer at each stage to understand disease progression.

Leaf spot typically manifests as multiple small spots on the leaves, with colors varying from dark brown, to yellow, to black, and the spots differ in size and shape. As the disease progresses, the number of leaf spots may increase rapidly, leading to an overall mottled appearance that affects the efficiency of photosynthesis, further impacting the growth and development of wheat. To capture this progression, we recorded the appearance of leaf spots from the jointing to grain filling stages, paying particular attention to the spread and impact of the spots at each stage. Glume blotch is more pronounced, usually causing the entire spike to wither, becoming dry and brittle. Wheat affected by glume blotch often shows grains that appear grayish-white or brown, a color change resulting from the pathogen’s invasion of the wheat tissue. When collecting these samples, we primarily focused on the heading and grain filling stages, observing and recording the morphological changes in spikes and their surrounding plants during this period of peak infection.

Rust appears as rust-colored spots on leaves and stems, typically displaying bright orange or brown colors. In severe cases, it can hinder the overall growth of the plant. The presence of rust not only affects photosynthesis but may also suppress wheat growth. For rust, we captured its progression from the jointing to grain filling stages, carefully documenting the distribution, density, and impact of rust spots on photosynthesis and overall plant health. These systematic observations across different growth stages ensured that the collected samples represented the full lifecycle of wheat diseases, providing comprehensive and diverse data for model training.

#### 3.1.2. Wheat Spike Counting Dataset Collection

A total of 2784 images were used for the wheat spike counting task. The specific collection method involved several steps. First, images were captured in the field at different growth stages of wheat, covering periods from heading to grain filling, to ensure diversity in spike size, shape, and distribution. The selected fields provided a range of environmental conditions, including variations in light and plant density, ensuring the dataset reflects real-world scenarios. Under suitable lighting conditions, we used a tripod to stabilize the camera, ensuring image stability during shooting. To avoid influences from changes in the external environment, we typically conducted photography in the early morning or late afternoon when the lighting is softer, conducive to capturing details. All images were manually annotated by agricultural experts to ensure accurate labeling of wheat spikes. Annotation was performed using a dedicated labeling tool, with a focus on delineating individual spikes in both dense and sparse areas. To enhance the dataset’s diversity, the images include top-down and side-view perspectives, with resolutions suitable for precise spike counting tasks.

#### 3.1.3. Image Enhancement

**Figure 2:**
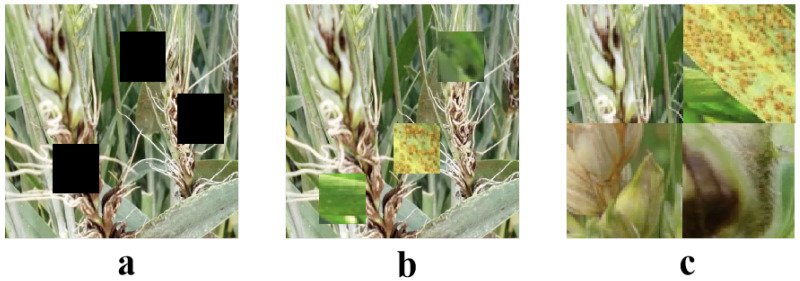
Dataset augmentation. (**a**) is CutOut, (**b**) is CutMix, (**c**) is Mosaic.

In this study, not only did we use basic image enhancement methods such as random rotation, flipping, scaling, cropping, and color adjustment, but we also introduced techniques like CutOut, CutMix, and Mosaic, which have been proven to effectively enhance the performance of deep learning models in visual tasks, as shown in Figure 2.

CutOut is a relatively simple yet effective data augmentation method that enhances the diversity of the training data by randomly removing parts of the image. The basic idea of this method is to simulate object occlusion, forcing the model to recover information from incomplete inputs. In implementing CutOut, we first define a fixed-size rectangle and randomly choose a location on the image to set its pixel values to zero. Mathematically, assuming the rectangle size is h×w and the image size is H×W, the position (x,y) of the rectangle can be described as follows:

(5) x∼Uniform(0,W−w),y∼Uniform(0,H−h)

Then, all pixel values within this rectangular area are set to zero to simulate the occlusion effect. CutMix further develops the concept of data augmentation, not only operating within a single image but also involving region exchange between two images. Specifically, CutMix cuts out a rectangular region from one image and pastes it into the same position in another image. This method not only increases the complexity of the scene but also promotes the model’s ability to handle partial occlusions and incomplete information. The operation of CutMix can be expressed through the following mathematical formula:

(6) $$\left(x,y,w,h\right)=\mathrm{bbox}\left(I_{A}\right),\;I_{B}\left[x:x+w,y:y+h\right]=I_{A}\left[x:x+w,y:y+h\right]$$

where $I_{A}$ and $I_{B}$ are the two images selected for the CutMix operation, and (x,y,w,h) define the position and size of the rectangular region cut from $I_{A}$ and replaced in $I_{B}$. Mosaic is a more complex data augmentation technique that stitches four different images into one new image. The main advantage of this method is that it can simulate the simultaneous presence of multiple objects in a single image, thus enhancing the model’s ability to handle complex scenes. In practice, we usually randomly select four training images, crop them to the same size, and then stitch these four parts together to form a new large image. If each image is cropped to H/2×W/2, the stitching operation can be represented as follows:

(7) $$I_{\mathrm{mosaic}}=\left[\begin{array}{cc} I_{A} & I_{B}\\ I_{C} & I_{D} \end{array}\right]$$

where $I_{A},I_{B},I_{C},I_{D}$ are the four selected images. These image enhancement techniques allow our model to better adapt to various challenges in practical applications, such as different lighting conditions, complex backgrounds, and partial occlusions. These techniques not only enhance the diversity of the data but also help the model learn to extract key features from incomplete or complex visual information, thereby achieving better performance in actual tasks of wheat spike counting and disease detection.

### 3.2. Dataset Construction

#### 3.2.1. Dataset Annotation

In this study, we used the LabelMe tool for image annotation. The primary task of image annotation is to accurately identify and label the targets of interest in the images, which are mainly wheat spikes in this case. Each spike is marked with a bounding box, typically defined as a rectangular area that surrounds the target object (wheat spike) and excludes as much of the background as possible. Mathematically, the bounding box can be represented as follows:

(8) $$B=(x_{\min},y_{\min},x_{\max},y_{\max})$$

where $\left(x_{\min},y_{\min}\right)$ and $\left(x_{\max},y_{\max}\right)$ are the coordinates of the top-left and bottom-right corners of the bounding box in the image coordinate system, respectively. This representation method is concise and efficient, suitable for object detection algorithms in computer vision. To ensure the accuracy and consistency of data annotation, we adopted a multi-step verification and correction mechanism. Initially, all annotators received training on how to use the LabelMe tool and how to identify and mark wheat spikes and diseases. During the annotation process, each image was independently completed by at least two annotators, followed by a review from an agricultural expert. This multi-level annotation and review process effectively reduces human errors and subjective judgment differences. The accuracy of the annotations not only affects the effectiveness of model training but also directly relates to the model’s performance in practical applications. For this, we also introduced a series of mathematical metrics to assess and optimize the quality of the annotations, for example, using the Intersection over Union (IoU) to measure the consistency of bounding boxes between different annotators:

(9) $$\mathrm{IoU}\left(B_{i},B_{j}\right)=\frac{\mathrm{area}(B_{i}\cap B_{j})}{\mathrm{area}(B_{i}\cup B_{j})}$$

where $B_{i}$ and $B_{j}$ are the bounding boxes of the same wheat spike annotated by different annotators. The higher the IoU value, the better the consistency of the annotation. In addition to the position and size of the spikes, marking diseases is also an important part of data annotation. The disease marking requires not only identifying the affected spikes but also assessing the severity of the disease. For this purpose, we assigned a disease level to each spike marked with a disease, based on the impact of the disease on the appearance of the spike. Mathematically, the disease level can be represented by a continuous scalar *v*:

(10) v∈[0,1]

Here, 0 indicates no disease and 1 indicates extremely severe disease. This quantification method helps the model to learn and predict the impact of diseases more finely.

#### 3.2.2. Dataset Partition

To ensure the stability and reliability of the model on different data, reasonable dataset division and verification methods are crucial. In this study, the dataset was divided into training, validation, and test sets in an 8:1:1 ratio. The training set is used for learning and training the model, the validation set mainly for adjusting model parameters and preventing overfitting, and the test set for evaluating the final performance of the model.

Moreover, for k-fold cross-validation, the entire dataset is usually evenly split into *k* subsets, each time using k−1 subsets as training data and the remaining one as test data. This process is repeated *k* times, each time selecting a different subset as the test data. In this study, 5-fold cross-validation was used, i.e., k=5. The advantage of this method is that it allows all observation data to be used both for training and testing, thereby providing a more comprehensive evaluation of model performance. Through the aforementioned dataset division and verification methods, it is possible to effectively control the various variables in the model training process, ensuring that the model performs more stably and reliably on unseen data.

### 3.3. Proposed Method

#### 3.3.1. Overview

In the methodology construction process of this paper, we designed a wheat spike counting and disease detection model that integrates object detection and a probability density-based attention mechanism. After data preprocessing, the image first enters the feature extraction module. In this module, a CNN performs preliminary feature extraction on the image, generating feature maps. The output of the feature channel processing is a high-dimensional feature vector $V_{i}$, where each feature vector $V_{i}$ represents the features of the wheat spike or disease region in the *i*-th area, providing a foundation for subsequent classification and regression. The feature vectors $V_{i}$ outputted by the feature channel processing module then enter the Transformer module. In this module, the model uses the self-attention mechanism to further strengthen feature relationships, especially capturing long-range dependencies within large image regions. Once the Transformer module completes its processing, the model moves into the probability density-based attention mechanism module. This module guides the model to focus on densely populated wheat spike regions by calculating the probability density map within each region. The output from this module is a weighted feature map, which significantly enhances the model’s detection capability in densely populated wheat spike areas. The final part of the model is the density loss function module. Specifically designed for wheat spike counting and disease detection, this module optimizes the model’s detection performance in dense regions. By comparing the predicted density map with the true density map, the density loss function optimizes the model to improve detection accuracy. This design enables the model to better adapt to complex farmland scenes, achieving higher counting accuracy and detection precision in densely populated wheat spike areas.

#### 3.3.2. Transformer Model for Object Detection Tasks

**Figure 3:**
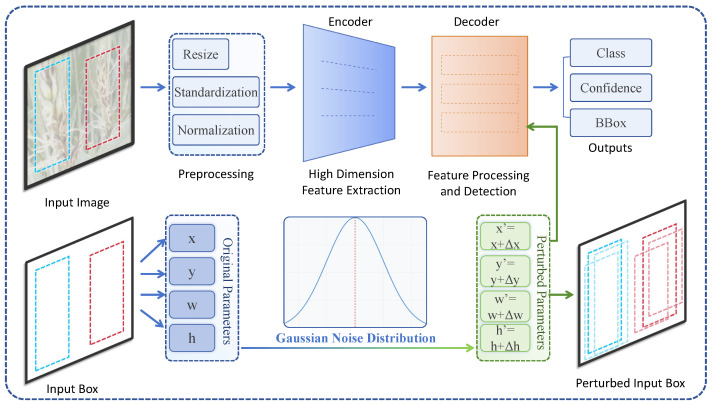
The diagram illustrates the workflow of an object detection task using a Transformer model. Starting from the input image, it details the three main stages: data preprocessing, encoder processing, and decoder processing.

This paper adopts a Transformer model structure based on object detection tasks, which combines a self-attention mechanism with an encoder–decoder structure. It is particularly suitable for large-scale agricultural images for wheat spike counting and disease detection tasks. The core idea is to capture long-range dependencies through a self-attention mechanism, enabling precise recognition and localization of wheat spikes and disease regions, as shown in Figure 3.

**Figure 4:**
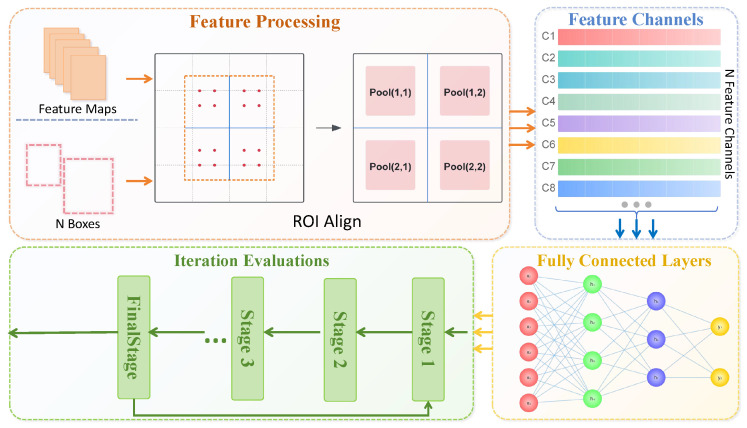
The diagram shows the detailed design of the object detection network structure. It starts with feature processing, extracts and aligns feature maps through the ROI Align module, and then processes them in different feature channels, including the setup of fully connected layers. N Boxes: refers to the number of proposed regions or bounding boxes in an image; $x_{i}$: refers to elements in weithts.

First, the feature map is passed to the Transformer encoder, which utilizes the self-attention mechanism to further extract global contextual information. During encoding, each region of the input image generates a corresponding feature representation, allowing the model to perform well in identifying occluded and densely packed wheat spikes. In the encoder structure, each layer contains a multi-head self-attention mechanism and a feedforward neural network, where feature representations are weighted in the multi-head self-attention layer. Each attention head operates independently, followed by concatenation and linear transformation to obtain the final feature representation. This multi-head mechanism can capture wheat spike features of different scales and locations within the image. The encoded feature map is then input into the decoder to detect the target objects, as shown in Figure 4.

The decoder uses interaction between the input features and query vectors to generate output class and bounding box information. Specifically, in each layer, the decoder first interacts between the query vectors and encoded features, updating the query vectors using the self-attention mechanism. It then combines the encoder’s output context features with the query through cross-attention to form precise target localization. At this stage, the model continuously adjusts the representation of the query vectors, allowing each query vector to obtain appropriate feature values at different spatial positions. This design leverages the advantages of multi-scale feature representation and long-range dependencies, enhancing the model’s adaptability to densely packed and occluded wheat spike regions. Next, the output feature map is weighted by the density-based attention mechanism to further focus on high-density wheat spike areas. By combining the self-attention mechanism with a probability density-weighted approach, the model prioritizes target regions in the feature space, improving accuracy in wheat spike counting and disease detection.

Designing such a Transformer-based model offers several advantages. Firstly, the Transformer model captures long-range dependencies in wheat field images, enabling strong performance on large-scale farmland images. Secondly, the encoder–decoder structure allows for the extraction of global features and progressive focusing when performing object detection tasks, making it well suited to complex scenes. Through the multi-head self-attention mechanism, the model can capture distinct features at various locations, ensuring sensitivity to each individual wheat spike in densely populated scenes and improving detection accuracy.

#### 3.3.3. Probability Density-Based Attention Mechanism

**Figure 5:**
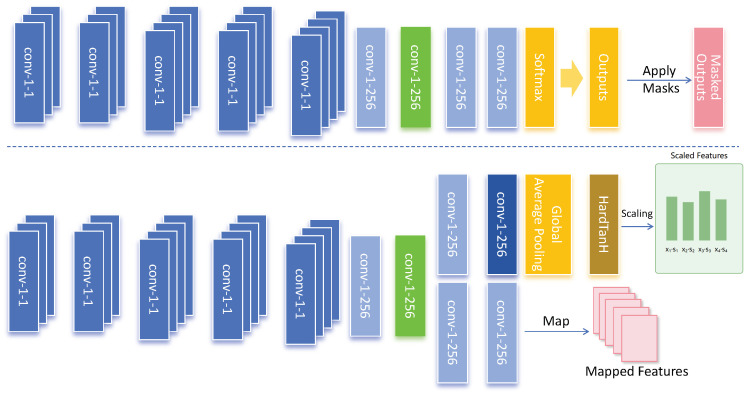
This schematic shows the specific implementation structure of the attention mechanism based on probability density. Each module in the diagram represents the various computational steps from input to output, including convolution layers, global average pooling, and final output processing.

In the model proposed in this paper, the probability density-based attention mechanism plays a crucial role in feature extraction and regional focusing. Compared to the traditional self-attention mechanism in Transformers, this mechanism enhances the model’s focus on high-density wheat spike regions through probability density estimation, as shown in Figure 5.

The traditional self-attention mechanism in Transformers mainly relies on similarity calculations between the query (*Q*), key (*K*), and value (*V*) matrices to distribute weights. However, in wheat spike counting and disease detection tasks, wheat spike distribution in the field is often uneven, with significant density differences between wheat spikes. The conventional self-attention mechanism struggles to effectively handle this density variation. Therefore, we designed a probability density-based attention mechanism that uses density estimation to guide the model’s focus on densely packed wheat spike regions.

In the probability density-based attention mechanism, we first generate a density map D(x,y) through density estimation, where each position (x,y)’s density value indicates the density level of wheat spikes in that region. Based on the density map, we use a weighting mechanism to generate an attention weight α(x,y) that guides the model to focus on high-density areas.

In the specific network design of this attention module, we used the following parameter configuration:

1. Convolutional layer settings: - The initial convolutional layer is ‘conv-1-1’, which reduces the dimensionality of the input feature map to generate an initial feature representation. - The middle convolutional layer is configured as ‘conv-1-256’, responsible for extracting density-related features, with an output channel of 256 to enrich feature representation capability. - The final convolutional layer is ‘conv-1-1’, which again reduces the dimensionality to match the density map.
2. Global average pooling: The global average pooling layer extracts global information from the feature map and combines it with density weights to generate a feature representation adapted to the global context.
3. Activation function: The HardTanH activation function is used to perform a nonlinear transformation on the features, enhancing the model’s robustness.
4. Skip connection: A skip connection structure is applied between each convolutional layer, allowing shallow information to merge with deep features and retain more detailed features.

In the probability density-based attention mechanism, we introduced a shortcut structure to enhance information transfer between different layers. The shortcut structure allows the model to retain low-level feature information in a multi-layer convolutional network, thus incorporating attention to the original features into high-level features. The mathematical expression for this is as follows:

(11) $$F_{\mathrm{shortcut}}\left(x,y\right)=\lambda \cdot F_{\mathrm{low}}\left(x,y\right)+\left(1-\lambda \right)\cdot F_{\mathrm{high}}\left(x,y\right)$$

where $F_{\mathrm{low}}\left(x,y\right)$ and $F_{\mathrm{high}}\left(x,y\right)$ represent the low-level and high-level features, respectively, and λ is a learnable coefficient that controls the fusion ratio of low-level and high-level features. Through the skip attention mechanism, the model can merge shallow information into deep features, enhancing the detail in feature representation.

The advantage of the probability density-based attention mechanism and the shortcut structure in this task is that they can adaptively adjust the attention focus, making the model more sensitive to dense wheat spike regions. The mathematical proof is as follows:

Let the feature map F(x,y), after passing through the skip attention mechanism, yield an output of $F_{\mathrm{output}}\left(x,y\right)$; then,

(12) $$F_{\mathrm{output}}\left(x,y\right)=\alpha \left(x,y\right)\cdot F_{\mathrm{skip}}\left(x,y\right)$$

where α(x,y) is the attention weight based on the density map and $F_{\mathrm{skip}}\left(x,y\right)$ is the fused skip feature. This demonstrates that $F_{\mathrm{output}}\left(x,y\right)$ receives a higher weight in densely populated wheat spike regions, allowing the model to better capture the details of these areas.

#### 3.3.4. Density Loss Function

Traditional loss functions mainly include mean squared error (MSE) and cross-entropy loss. MSE is typically used in regression tasks, measuring the accuracy of predictions by calculating the squared error between predicted and actual values, defined as follows:

(13) $$\mathrm{MSE}=\frac{1}{N}\sum_{i=1}^{N}{\left(y_{i}-{\hat{y}}_{i}\right)}^{2}$$

where $y_{i}$ and ${\hat{y}}_{i}$ represent the true and predicted values of the *i*-th sample, respectively. However, MSE is sensitive to noise and outliers, and, in dense object detection tasks, it often leads to imbalance issues. When wheat spikes are unevenly distributed or occluded, MSE performs poorly because it fails to account for target density information. The cross-entropy loss function performs well in classification tasks, and is expressed as follows:

(14) $$\mathrm{Cross}\;\mathrm{Entropy}=-\sum_{i=1}^{N}y_{i}\log\left({\hat{y}}_{i}\right)$$

Although cross-entropy works well in object detection, relying solely on classification results with cross-entropy loss cannot ensure accurate prediction in dense target regions or under occlusion, leading to cumulative errors in counting tasks. To overcome the limitations of traditional loss functions, we propose a density loss function that integrates density information of targets to optimize the model’s counting performance. Specifically designed for dense object detection tasks, this density loss function adjusts the loss weights through density information, enabling the model to achieve higher precision in densely populated wheat spike regions. The density loss function is defined as follows:

(15) $$L_{\mathrm{density}}=\frac{1}{N}\sum_{i=1}^{N}\left(∥D_{\mathrm{pred}}\left(i\right)-D_{\mathrm{true}}{\left(i\right)∥}^{2}+\lambda \sum_{(x,y)\in S}\alpha \left(x,y\right)\cdot {∥D_{\mathrm{pred}}\left(x,y\right)-D_{\mathrm{true}}\left(x,y\right)∥}^{2}\right)$$

where $D_{\mathrm{pred}}\left(i\right)$ and $D_{\mathrm{true}}\left(i\right)$ denote the predicted and true density values at the *i*-th position, *S* represents the pixel set of the dense region, λ is a regularization coefficient controlling the weight for dense regions, and α(x,y) is the attention weight based on the density map, used to weight the loss for dense areas. The density loss function achieves weighted focus on target density through two main components: the squared error between predicted and actual density maps, and the weighted error for dense regions. The first component ensures the model’s overall density estimation across regions, while the second component particularly focuses on accuracy within dense regions. The weight α(x,y) for dense regions is defined as follows:

(16) $$\alpha \left(x,y\right)=\frac{\exp(D_{\mathrm{true}}\left(x,y\right))}{\sum_{(x^{\prime },y^{\prime })\in S}\exp\left(D_{\mathrm{true}}\left(x^{\prime },y^{\prime }\right)\right)}$$

The mathematical rationale behind this design is that pixels in dense regions have higher density values, hence receive greater weight in the loss calculation. In this way, the model prioritizes optimization of predictions in dense areas, allowing it to focus on wheat spike-dense regions and thereby improve counting accuracy. By introducing a weighted squared error in dense regions, the density loss function enables the model to adaptively adjust its attention to high-density areas during optimization. We prove that this loss function converges to the optimal density estimation as follows. Assuming the objective is to minimize the density loss function $L_{\mathrm{density}}$, the gradient is given by the following:

(17) $$\frac{\partial L_{\mathrm{density}}}{\partial D_{\mathrm{pred}}\left(x,y\right)}=2\left(D_{\mathrm{pred}}\left(x,y\right)-D_{\mathrm{true}}\left(x,y\right)\right)+2\lambda \alpha \left(x,y\right)\cdot \left(D_{\mathrm{pred}}\left(x,y\right)-D_{\mathrm{true}}\left(x,y\right)\right)$$

As shown, the gradient magnitude is directly proportional to the difference between predicted and true densities, ensuring the model reduces error progressively during training, with the dense region weight α(x,y) accelerating convergence in these areas. The advantage of the density loss function lies in its ability to effectively handle dense region object detection and counting problems. In wheat spike counting tasks, wheat spikes are often densely distributed and occluded, which traditional loss functions cannot fully address. By introducing the density loss function, the model can automatically identify high-density regions and weight them accordingly, ensuring high detection precision in dense areas.

Furthermore, the density loss function incorporates probability density-based attention weight α(x,y) for dense areas, achieving mathematically weighted attention to high-density regions within the loss layer. This design not only improves the model’s overall robustness but also provides significant advantages in handling dense scenes and occlusion issues. Compared to traditional mean squared error and cross-entropy loss functions, the density loss function achieves enhanced real-world performance through more refined weight control, particularly excelling in wheat field scenarios.

### 3.4. Experimental Setup

#### 3.4.1. Evaluation Metrics

In this study, to comprehensively evaluate the model’s performance in wheat spike counting and disease detection tasks, we selected several key performance metrics: precision, recall, accuracy, and mAP. Precision primarily measures the proportion of actual positives among the positives identified by the model, which is crucial for assessing the model’s accuracy in predicting wheat spikes or diseases. Recall measures the proportion of actual positives that the model correctly identifies. In wheat spike counting and disease detection, a high recall means the model minimally misses any significant detection targets, thus ensuring that all potential issues are addressed. Accuracy provides the overall correctness rate of the model’s predictions, including both positives and negatives, and is the most intuitive performance metric, directly reflecting the model’s performance across the entire dataset. Mean average precision is a more complex and comprehensive metric that considers the average precision of different categories and calculates the mean across the dataset. This metric is particularly important in multi-category tasks of wheat spike and disease detection because it reflects the model’s capability to handle various situations. Their mathematical formulas are as follows:

(18) $$\mathrm{Precision}=\frac{TP}{TP+FP}$$

(19) $$\mathrm{Recall}=\frac{TP}{TP+FN}$$

(20) $$\mathrm{Accuracy}=\frac{TP+TN}{TP+TN+FP+FN}$$

(21) $$\mathrm{mAP}=\frac{1}{Q}\sum_{q=1}^{Q}{\mathrm{AP}}_{q}$$

Here, TP (True Positive) is the number of positives correctly identified by the model, FP (False Positive) is the number of negatives incorrectly marked as positive, FN (False Negative) is the number of positives missed, and TN (True Negative) is the number of negatives correctly identified, *Q* is the number of categories, and ${\mathrm{AP}}_{q}$ is the average precision for category *q*.

#### 3.4.2. Hardware and Software Platform

This study selected a high-performance computing platform equipped with the NVIDIA Tesla V100 GPU to ensure that all experimental activities could proceed efficiently and stably. The NVIDIA Tesla V100 GPU, designed specifically for deep learning and high-performance computing (HPC), is based on NVIDIA’s Volta GPU architecture and offers exceptional computational power and efficiency. The Tesla V100 GPU features 5120 CUDA cores and 640 Tensor cores, the latter being specially optimized for deep learning training and inference performance. Using this GPU significantly accelerates the model training process, especially when handling large-scale image data and complex model computations. Additionally, our computing platform is equipped with high-speed CPU processors and substantial RAM to support the GPU’s computational and data handling needs. Specifically, the platform includes multi-core Intel Xeon CPUs and sufficient memory (usually over 256 GB), ensuring that the system’s response speed and data processing capabilities meet the requirements during large-scale data loading, preprocessing, and model training.

On the software side, we utilized the mainstream deep learning framework PyTorch. To fully leverage the performance of the Tesla V100 GPU, we specially optimized the framework’s configuration, including installing the latest GPU drivers and CUDA libraries, along with the corresponding cuDNN library, which is NVIDIA’s GPU-accelerated library for deep neural networks. During the experiments, we also used various data management and model training auxiliary tools, such as Docker container technology and Kubernetes cluster management, which help us efficiently manage the experimental environment and ensure the reproducibility of the experiments.

#### 3.4.3. Optimizer and Hyperparameter Settings

In this study, to optimize the model training process, we chose the Adam optimizer for parameter optimization, with an initial learning rate set at 0.001. To address the issue of overfitting, we also introduced a learning rate decay mechanism and early stopping techniques to ensure that the model could maintain performance while avoiding excessive fitting to the training data. Additionally, through multiple rounds of experimentation, we determined that the batch size should be set to 32, and the number of iterations adjusted dynamically based on the performance on the training set, typically stopping when the model’s performance no longer improved or when the error on the validation set began to increase. These settings helped us effectively balance the model’s learning efficiency and resource usage, achieving optimal training results.

#### 3.4.4. Baseline

In this paper, to evaluate the effectiveness of our proposed model, we compared it with several existing advanced baseline models, including YOLOv8 [48], YOLOv9 [49], Tiny-Segformer [18], DETR (Detection Transformer) [50], and WheatNet [51]. These models are representative in the field of object detection, and their performances provide a practical benchmark for our model. The YOLO models simplify the object detection task into a single regression problem, mapping directly from image pixels to bounding box coordinates and class probabilities. The core idea is to use the entire image as input and predict the category and location of objects through a single forward pass. YOLOv8 and YOLOv9 build on this foundation, incorporating additional network layers and advanced feature extraction technologies to enhance detection accuracy and speed.

Tiny-Segformer is a neural network designed specifically for lightweight scenarios, combining the self-attention mechanism of Transformers with a lightweight segmentation strategy. This model optimizes the structure of Transformers to reduce computational load while maintaining robust feature extraction capabilities. Tiny-Segformer is particularly suitable for processing large-scale image data, achieving rapid and precise image segmentation through its efficient encoder–decoder structure. DETR is a novel object detection framework that uses the structure of Transformers to directly predict the classes and bounding boxes of objects. WheatNet is a network specifically designed for wheat spike detection, optimizing the recognition and localization of wheat spikes by integrating deep learning and image processing technologies.

By comparing these baseline models with our model based on a probability density-based attention mechanism, we are able to accurately assess the latter’s performance and advantages in wheat spike counting and disease detection tasks. Specifically, our model’s ability to handle dense areas and complex backgrounds, as well as its capability to maintain high-speed processing while providing high-precision detection, are important criteria for assessment. Through this systematic comparison, the practicality and effectiveness of our proposed model can be further validated.

## 4. Results and Discussion

### 4.1. Disease Detection Results

The design of this experiment aims to evaluate the performance of different models in the task of disease detection, thereby validating the effectiveness and advantages of the proposed model. By comparing various existing models, including YOLOv8, Tiny-Segformer, DETR, WheatNet, and YOLOv9, as well as the model based on the probability density attention mechanism introduced in this paper, we analyze their performance based on key indicators such as precision, recall, accuracy, and mAP, as shown in Table 2.

From the experimental results, it can be observed that YOLOv8 has a precision of 0.81, a recall of 0.79, an accuracy of 0.80, and an mAP of 0.80, demonstrating relatively good baseline performance. The indicators for Tiny-Segformer show a slight improvement, with a precision of 0.85, a recall of 0.82, an accuracy of 0.83, and an mAP of 0.82. This improvement is attributed to its lightweight design that combines the self-attention mechanism and segmentation strategy, enhancing its performance in image segmentation tasks. The DETR model further improves performance, achieving a precision of 0.87, a recall of 0.84, an accuracy of 0.85, and an mAP of 0.84, thanks to its use of a global attention mechanism that can effectively capture long-range dependencies. WheatNet, specifically designed for wheat spike detection, performed excellently with a precision of 0.89, a recall of 0.87, an accuracy of 0.88, and an mAP of 0.87, indicating its strong adaptability in specific agricultural scenarios. YOLOv9 achieved the best performance across all metrics, with a precision of 0.91, a recall of 0.88, an accuracy of 0.90, and an mAP of 0.89, showcasing the advantages of its optimized network structure for real-time detection. Finally, the performance of the proposed method surpassed all other models, achieving a precision of 0.93, a recall of 0.89, an accuracy of 0.91, and an mAP of 0.90. This indicates that the design based on the probability density attention mechanism effectively enhances the overall performance of the model in disease detection. Analyzing the characteristics of different models reveals that the YOLO series has an advantage in processing speed, making it particularly suitable for real-time applications. However, it may face limitations when dealing with high-density disease detection in complex backgrounds. Tiny-Segformer improves feature extraction capabilities through its self-attention mechanism, making it suitable for more detailed image segmentation tasks. DETR’s ability to capture global features gives it a stronger adaptability in complex scenarios. WheatNet, designed specifically to meet agricultural needs, also performs exceptionally well. Our model mathematically incorporates a probability density attention mechanism, providing clear advantages in feature extraction and attention adjustment in dense regions. This mechanism better addresses challenges related to occlusion and background complexity, enhancing detection accuracy. Therefore, the experimental results not only reflect the accuracy of the models but also demonstrate the applicability and effectiveness of each model in different tasks, offering valuable references for future research and practical applications.

### 4.2. Wheat Spike Counting Experimental Results

The design of this experiment aims to evaluate the performance of different models in the task of wheat spike counting, thereby validating the effectiveness and superiority of the proposed model. By comparing several existing advanced models, we can gain a comprehensive understanding of their performance in the wheat spike counting task, as shown in Table 3.

From the experimental results, it can be observed that YOLOv8 achieved a precision of 0.83, a recall of 0.80, an accuracy of 0.81, and an mAP of 0.81, demonstrating a solid baseline performance. As a real-time detection model, YOLOv8 has advantages in processing speed, but its performance may be limited when dealing with complex wheat spike distributions. The indicators for Tiny-Segformer show an improvement, with a precision of 0.86, a recall of 0.83, an accuracy of 0.84, and an mAP of 0.83. This improvement is attributed to its design that combines the self-attention mechanism and segmentation strategy, providing strong feature extraction capabilities for image segmentation tasks. The performance of the DETR model further increased, achieving a precision of 0.88, a recall of 0.85, an accuracy of 0.86, and an mAP of 0.85, indicating its effectiveness in capturing global features when processing complex scenes. YOLOv9 performed excellently across all metrics, with a precision of 0.89, a recall of 0.85, an accuracy of 0.87, and an mAP of 0.88, showcasing the advantages of its optimized network structure for real-time detection. WheatNet, specifically designed for wheat spike detection, demonstrated a remarkable performance with a precision of 0.90, a recall of 0.87, an accuracy of 0.89, and an mAP of 0.88, indicating its strong adaptability in specific agricultural scenarios. Finally, the proposed method outperformed all other models, achieving a precision of 0.91, a recall of 0.88, an accuracy of 0.90, and an mAP of 0.90. This demonstrates that the design based on the probability density attention mechanism effectively enhances the overall performance of the model. Analyzing the characteristics of different models reveals that the YOLO series has advantages in processing speed, making them particularly suitable for real-time applications. However, they may face limitations when dealing with high-density wheat spikes and complex backgrounds. Tiny-Segformer improves detail capture capabilities through its optimized Transformer structure, making it well suited for more complex image segmentation tasks. The global feature capture ability of DETR gives it a stronger adaptability in complex scenarios. WheatNet performs exceptionally well due to its specialized design to meet agricultural needs. Our model mathematically incorporates a probability density attention mechanism, which provides clear advantages in feature extraction and attention adjustment in dense areas. Overall, the results reflect the accuracy of the models and demonstrate their applicability and effectiveness in different tasks, providing valuable references for future research and practical applications.

### 4.3. Ablation Experiment on Different Attention Mechanisms

The design of this experiment aims to explore the effects of different attention mechanisms on disease detection and wheat spike counting tasks, thereby validating the superiority of the probability density-based attention mechanism.

From Table 4, it can be seen that, in the disease detection task, the model using the standard self-attention mechanism performed relatively poorly, with a precision of 0.75, a recall of 0.70, an accuracy of 0.73, and an mAP of 0.73. This indicates that traditional self-attention mechanisms have certain limitations in feature extraction and focusing capabilities. After introducing the convolutional block attention module, the model’s performance improved significantly, with precision rising to 0.85, recall at 0.82, accuracy at 0.83, and mAP at 0.84. This demonstrates that the convolutional block attention module can more effectively focus on key areas when handling local features, thus improving the model’s ability to detect diseases. In contrast, the model using the probability density attention mechanism achieved the best performance, with a precision of 0.93, a recall of 0.89, an accuracy of 0.91, and an mAP of 0.90, showcasing the mechanism’s superiority in focusing on high-density areas. In the wheat spike counting task, the standard self-attention mechanism also showed low performance, with precision at 0.72, recall at 0.70, accuracy at 0.71, and mAP at 0.71. The convolutional block attention module improved these metrics to a precision of 0.83 and recall of 0.80, indicating its advantages in image feature extraction. The probability density attention mechanism again demonstrated strong performance, with precision at 0.91, recall at 0.88, accuracy at 0.90, and mAP at 0.90, further validating its effectiveness in the wheat spike counting task.

The standard self-attention mechanism primarily relies on the direct relationships between input features, failing to adequately consider the density information of target areas, which may lead to information loss when dealing with complex scenes. The convolutional block attention module improves the model’s sensitivity to important areas by focusing on local features but still struggles to perform well in dense regions. In contrast, the probability density attention mechanism introduces guidance from the density map, allowing the model to better adjust its focus, thus achieving higher weights in disease and wheat spike dense areas, resulting in more precise feature extraction and more effective detection capabilities. This design fully leverages the density information in images, enabling the model to adaptively focus on key information regions, thereby exhibiting a superior performance in practical applications.

### 4.4. Ablation Experiment on Different Loss Functions

The design of this experiment aims to evaluate the effects of different loss functions on disease detection and wheat spike counting tasks, thereby validating the advantages of the proposed density loss function. By comparing models using cross-entropy loss, focal loss, and density loss, we can gain a comprehensive understanding of the performance and applicability of various loss functions in handling specific tasks. The main metrics used in the experiment include precision, recall, accuracy, and mean average precision (mAP), which effectively reflect the model’s performance in practical applications.

From Table 5, it can be seen that, in the disease detection task, the model using cross-entropy loss performed relatively poorly, with a precision of 0.75, a recall of 0.72, an accuracy of 0.73, and an mAP of 0.73. The cross-entropy loss function may lead to insufficient recognition capability for minority classes when facing class imbalance or dense regions, thus limiting its performance. In contrast, the model using focal loss showed improvements across all metrics, achieving a precision of 0.86, a recall of 0.83, an accuracy of 0.84, and an mAP of 0.84. Focal loss effectively enhances the model’s performance in dense areas by assigning higher weights to hard-to-classify samples. This loss function is particularly suitable for addressing class imbalance issues, helping the model focus better on samples that may otherwise be overlooked. In the case of the model using density loss, the results were even more outstanding, with a precision of 0.93, a recall of 0.89, an accuracy of 0.91, and an mAP of 0.90, demonstrating significant advantages in focusing on high-density areas and optimizing the overall performance of the model. In the wheat spike counting task, the model using cross-entropy loss similarly exhibited low performance, with a precision of 0.73, a recall of 0.70, an accuracy of 0.71, and an mAP of 0.71. However, after employing focal loss, the metrics improved significantly, reaching a precision of 0.87, a recall of 0.84, an accuracy of 0.85, and an mAP of 0.86, consistent with its performance in disease detection. Ultimately, the density loss also performed excellently in the wheat spike counting task, achieving a precision of 0.91, a recall of 0.88, an accuracy of 0.90, and an mAP of 0.90, further validating its effectiveness in handling dense targets.

Analyzing the experimental results, the cross-entropy loss function optimizes the model by calculating the logarithmic differences between predicted values and true values, which performs well in multi-class problems. However, in the presence of class imbalance, the model may struggle to learn from minority class targets. Focal loss introduces a modulation factor that effectively reduces the influence of easily classified samples, thereby strengthening the focus on hard-to-classify samples. This allows the model to capture critical information better when faced with complex backgrounds and occlusion situations. In contrast, the density loss function adjusts the loss weights by incorporating target density information, allowing the model to pay more attention to high-density areas, thus improving detection precision and recall. This design fully leverages the density characteristics present in the images, showcasing clear advantages for the model in specific tasks. Overall, this experiment not only demonstrates the impact of different loss functions on the model performance but also provides important theoretical foundations and practical guidance for future research and applications.

## 5. Conclusions

This study focuses on the important agricultural tasks of wheat spike counting and disease detection, highlighting the increasing significance of precision agriculture technologies in response to global concerns about food security. Traditional manual detection methods are not only time-consuming and labor-intensive, but also limited in accuracy and efficiency. By introducing deep learning techniques, especially in object detection and feature extraction for image processing, we can significantly enhance the precision of wheat spike counting and disease detection, providing a scientific basis for crop growth and management.

The main innovation of this paper lies in the proposal of a model based on the probability density attention mechanism. This model employs deep learning methods combined with advanced feature extraction strategies and attention mechanisms, particularly excelling in feature extraction and attention adjustment in dense regions. By comparing with several existing models, including YOLOv8, Tiny-Segformer, DETR, WheatNet, and YOLOv9, our model demonstrates excellent performance across key metrics. In the disease detection task, our model achieves a precision of 0.93, a recall of 0.89, an accuracy of 0.91, and an mAP of 0.90, clearly indicating the effectiveness of our approach. In the wheat spike counting task, the model also shows strong performance, with a precision of 0.91, a recall of 0.88, an accuracy of 0.90, and an mAP of 0.90, further validating its potential application in agricultural scenarios. Additionally, through ablation experiments on different loss functions, this study explores the impact of loss functions on the model performance. The results indicate that models using the density loss function excel in both disease detection and wheat spike counting tasks, with precision and recall significantly higher than those using cross-entropy loss and focal loss. The density loss function, by incorporating target density information, allows for more effective adjustment of the model’s focus, leading to improved performance in high-density areas. This design not only enhances the model’s accuracy but also provides robustness in complex backgrounds and occlusion situations. Overall, the research presented in this paper offers new ideas and methods for wheat spike counting and disease detection in precision agriculture.

## Figures and Tables

**Table 1:** Quantities of different disease data.

Disease	Quantity
Loose Smut	1210
Fusarium Head Blight	1394
Rust	1005
Powdery Mildew	1893
Leaf Spot	1976
Glume Blotch	1571

**Table 2:** Disease detection results.

Model	Precision	Recall	Accuracy	mAP
YOLOv8	0.81	0.79	0.80	0.80
Tiny-Segformer	0.85	0.82	0.83	0.82
DETR	0.87	0.84	0.85	0.84
WheatNet	0.89	0.87	0.88	0.87
YOLOv9	0.91	0.88	0.90	0.89
Proposed Method	0.93	0.89	0.91	0.90

**Table 3:** Counting accuracy results.

Model	Precision	Recall	Accuracy	mAP
YOLOv8	0.83	0.80	0.81	0.81
Tiny-Segformer	0.86	0.83	0.84	0.83
DETR	0.88	0.85	0.86	0.85
YOLOv9	0.89	0.85	0.87	0.88
WheatNet	0.90	0.87	0.89	0.88
Proposed Method	0.91	0.88	0.90	0.90

**Table 4:** Ablation experiment on different attention mechanisms.

Model	Precision	Recall	Accuracy	mAP
Disease Detection—Self-Attention	0.75	0.70	0.73	0.73
Disease Detection—CBAM	0.85	0.82	0.83	0.84
Disease Detection—Probability Density Attention	0.93	0.89	0.91	0.90
Counting—Self-Attention	0.72	0.70	0.71	0.71
Counting—CBAM	0.83	0.80	0.82	0.82
Counting—Probability Density Attention	0.91	0.88	0.90	0.90

**Table 5:** Ablation experiment on different loss functions.

Model	Precision	Recall	Accuracy	mAP
Disease Detection—Cross-Entropy Loss	0.75	0.72	0.73	0.73
Disease Detection—Focal Loss	0.86	0.83	0.84	0.84
Disease Detection—Density Loss	0.93	0.89	0.91	0.90
Counting—Cross-Entropy Loss	0.73	0.70	0.71	0.71
Counting—Focal Loss	0.87	0.84	0.85	0.86
Counting—Density Loss	0.91	0.88	0.90	0.90

## Data Availability

The data presented in this study are available on request from the corresponding author.
